# Dietary Acrylamide Intake and the Risk of Lymphatic Malignancies: The Netherlands Cohort Study on Diet and Cancer

**DOI:** 10.1371/journal.pone.0038016

**Published:** 2012-06-18

**Authors:** Mathilda L. Bongers, Janneke G. F. Hogervorst, Leo J. Schouten, R. Alexandra Goldbohm, Harry C. Schouten, Piet A. van den Brandt

**Affiliations:** 1 Department of Epidemiology, School for Oncology and Developmental Biology (GROW), Maastricht University Medical Centre +, Maastricht, The Netherlands; 2 Division Quality of Life, Department of Prevention and Health, Netherlands Organisation for Applied Scientific Research (TNO), Leiden, The Netherlands; 3 Division of Hematology, Department of Internal Medicine, Maastricht University Medical Centre +, Maastricht, The Netherlands; University of KwaZulu-Natal, South Africa

## Abstract

**Background:**

Acrylamide, a probable human carcinogen, is present in many everyday foods. Since the finding of its presence in foods in 2002, epidemiological studies have found some suggestive associations between dietary acrylamide exposure and the risk of various cancers. The aim of this prospective study is to investigate for the first time the association between dietary acrylamide intake and the risk of several histological subtypes of lymphatic malignancies.

**Methods:**

The Netherlands Cohort Study on diet and cancer includes 120,852 men and women followed-up since September 1986. The number of person years at risk was estimated by using a random sample of participants from the total cohort that was chosen at baseline (n  = 5,000). Acrylamide intake was estimated from a food frequency questionnaire combined with acrylamide data for Dutch foods. Hazard ratios (HRs) were calculated for acrylamide intake as a continuous variable as well as in categories (quintiles and tertiles), for men and women separately and for never-smokers, using multivariable-adjusted Cox proportional hazards models.

**Results:**

After 16.3 years of follow-up, 1,233 microscopically confirmed cases of lymphatic malignancies were available for multivariable-adjusted analysis. For multiple myeloma and follicular lymphoma, HRs for men were 1.14 (95% CI: 1.01, 1.27) and 1.28 (95% CI: 1.03, 1.61) per 10 µg acrylamide/day increment, respectively. For never-smoking men, the HR for multiple myeloma was 1.98 (95% CI: 1.38, 2.85). No associations were observed for women.

**Conclusion:**

We found indications that acrylamide may increase the risk of multiple myeloma and follicular lymphoma in men. This is the first epidemiological study to investigate the association between dietary acrylamide intake and the risk of lymphatic malignancies, and more research into these observed associations is warranted.

## Introduction

In 2002, the scientific world was alarmed by the discovery of acrylamide in foods by the Swedish Food Authority. Acrylamide was classified as a proven rodent carcinogen and a probable human carcinogen by the International Agency for Research on Cancer in 1994, because of its carcinogenicity in rodents and because of the similarity between the way it is metabolized in rodents and in humans [1]. Several frequently consumed foods, such as French fries, cookies and coffee, contain high levels of acrylamide [2]. Acrylamide in food is formed in Maillard browning reactions, in which amino acids, asparagine in particular, react with reducing sugars during baking or other thermal processing at temperatures higher than 120 degrees Celsius. Its formation depends on various cooking variables, particularly temperature and duration [3]. This causes large variations in the acrylamide content of different brands of the same food as well as among batches of a food of the same brand.

The mechanism by which acrylamide causes cancer in laboratory animals and by which it may cause cancer in humans is still unclear. Currently, the genotoxic action of glycidamide, which is an epoxide metabolite of acrylamide, is taken to be the mechanism of carcinogenic action in acrylamide risk assessments. Ample in vitro and in vivo animal studies have shown that acrylamide, mainly after metabolic conversion to glycidamide by the enzyme cytochrome P4502E1 (CYP2E1), causes chromosomal damage (aberrations, micronuclei, aneuploidy) and mutagenic effects [4]. However, the tissues with most DNA adducts or DNA mutations do not consistently correspond to the tissues in which cancer occurred in the rat studies [5], [6] and, more and more, other mechanisms of acrylamide carcinogenesis are being proposed [7], [8]. Animal studies have shown positive dose-response relations between acrylamide intake through drinking water and cancer in multiple organs in mice and rats, such as the mammary glands, thyroid gland, testes and the uterus [4]. The presence of these mainly sex hormone-related cancers in animals, suggests a hormonal pathway [4], [9], [10], perhaps occurring in addition to genotoxic effects.

Since the finding of the presence of acrylamide in foods in 2002, epidemiological studies have evaluated various cancer endpoints in association with dietary acrylamide exposure of humans. A positive association for endometrial cancer was observed in two prospective cohort studies [11], [12]. Both studies found a positive association for ovarian cancer as well, and in one of those studies this association was strongest in serous tumours [12]. Two studies, a cohort study and a nested case-control study, found a positive association between dietary acrylamide intake and the risk of estrogen receptor-positive breast cancer [13], [14]. Further, a positive association was observed with renal cancer risk [15], and with oral cavity cancer risk in non-smoking women [16], both in a Dutch cohort study. In a Finnish prospective cohort study, a positive association was observed with lung cancer risk in smoking men [17].

Some epidemiological studies found indications for inverse associations with cancer risk, such as with lung cancer risk in women [18], prostate cancer risk in never-smoking men, bladder cancer risk in women [15], and oro-and hypopharyngeal cancer risk in men [16].

Theoretically, every tissue in the human body, including lymphoid tissues, is a target for acrylamide carcinogenesis, because acrylamide is hydrophilic, and therefore it is able to diffuse passively throughout the whole body [19]. During the last decades, the occurrence of lymphatic malignancies has risen dramatically. This type of cancer is a heterogeneous group of malignancies derived from the T-cell and the B-cell development, and the most common types are multiple myeloma, diffuse large cell lymphoma and chronic lymphocytic leukemia [20]. Little is known about modifiable risk factors for these malignancies.

In the Finnish Alpha-Tocopherol, Beta-Carotene Cancer Prevention (ATBC) Study, no association was observed between dietary acrylamide intake and the risk of lymphomas in male smokers [17]. In this Finnish study, no analyses were done for histological subtypes of lymphatic malignancies, and therefore an association with a specific type of lymphatic malignancy might have been missed. Our study is the first to investigate the association between dietary acrylamide intake and the risk of several common subtypes of lymphatic malignancies.

## Methods

### Ethics Statement

By returning the completed questionnaire, the participants gave consent to participate in this study. The study protocol was approved by the Medical Ethics Committee of the University Hospital Maastricht and the Netherlands Organisation for Applied Scientific Research (TNO), division of Nutrition.

### Study Design and Population

In September 1986, 58,279 men and 62,573 women were enrolled in the Netherlands Cohort Study on diet and cancer (NLCS), a prospective cohort study [21]. All participants were at the age of 55–69 years at entry. Participants were selected through computerized municipal population registries.

Data processing and analysis were based on the case-cohort approach for efficiency reasons. The number of cancer cases (providing the numerator information for the estimation of cancer incidence rates) is the number of cases in the total cohort. The number of person-years at risk (providing the denominator information) was estimated by using a subcohort, a random sample of participants from the total cohort that was chosen at baseline (n  = 5,000). Subcohort members were censored when they died, emigrated, reached the end of follow-up, or became a case, whichever came first.

Participants in this study are considered to be a case if they had a microscopically verified diagnosis of a lymphatic malignancy during the 16.3 year follow-up period. Incident cases in the total cohort were detected by annual computerized record linkages to the Netherlands Cancer Registry and the Netherlands Pathology Registry. The completeness of cancer follow-up through linkages with these registries is estimated to be at least 96% [22]. The histological subtype of the lymphatic malignancy was coded by the Netherlands Cancer Registry, using the International Classification of Diseases for Oncology, adapted for the Netherlands [23]. These codes were used to reclassify the lymphatic malignancies in categories according to the WHO classification of tumours of hematopoietic and lymphoid tissues [24]. For cases that could not be assigned to a specific category, the summary of the pathology report of the Netherlands Pathology Registry was inspected, and, if possible, the case was assigned to a WHO category. Case numbers per histological subtype of lymphatic malignancies are given in table 1. The procedure is described elsewhere in more detail [25].

**Table 1:** Number of lymphatic malignancies in the Netherlands Cohort Study on diet and cancer (follow up: 16.3 years) according to the WHO classification.

Lymphatic malignancies	ICD-O-3 morphology codes	N^1^	N^2^
T-cell, all			54
T-cell lymphoma	9701–9709, 9714	41	
Mucosis fungoides	9700	20	
B-cell, precursor lesions			
Acute lymphocytic leukemia	9836–9837	12	
Lymphoblastic lymphoma	9727–9728	8	
Burkitt’s lymphoma	9687	4	
B-cell, other	9826, 9832–9833	2	
Malignant lymphomas, B-cell, mature neoplasms			
Diffuse large-cell lymphoma (DLCL)	9675, 9680, 9684	294	259
Follicular lymphoma (FL)	9690–9698	98	91
Waldenström macroglobulinemia and immunocytoma (WMI)	9671, 9761	90	89
Mantle cell lymphoma (MCL)	9673	63	56
Extranodal marginal B-cell lymphoma or MALT	9699	21	
Malignant lymphoma NOS	9590–9596	88	
Chronic lymphocytic leukemia	9670, 9823	224	200
Multiple myeloma	9731–9732, 9734	363	323
Hairy cell leukemia	9940	10	
Hodgkin lymphoma	9650–9669	37	
Total		1,375	1,233

Abbreviations: ICD-O-3, International Classification of Diseases for Oncology, 3^rd^ edition; MALT, mucosa-associated lymphoid tissue; NOS, not otherwise specified.

1 N after exclusion of prevalent cases at baseline.

2 N cases available for analyses, after exclusion of missing and inconsistent data. Only case numbers for subtypes with sufficient number of cases are given (so subgroups do not add up to 1,233).

**Figure 1:**
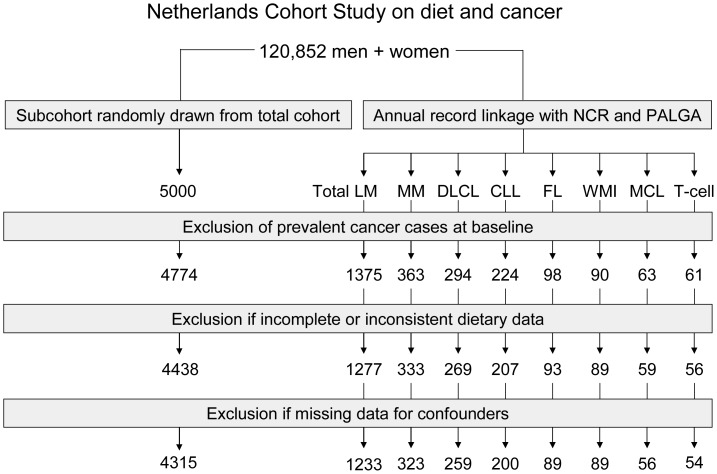
Flow diagram of subcohort members and cases used in the analysis. NCR  =  Netherlands Cancer Registry, PALGA  =  Netherlands Pathology Registry, LM  =  lymphatic malignancies, MM  =  multiple myeloma, DLCL  =  diffuse large cell lymphoma, CLL  =  chronic lymphocytic leukaemia, FL  =  follicular lymphoma, WMI  =  Waldenstrom macroglobulinemia and immunocytoma, MCL  =  mantle cell lymphoma, T-cell  =  T-cell lymphoma.

Cases and subcohort members were excluded from analysis if they reported cancer at baseline (except for skin cancer) or if their dietary data were incomplete or inconsistent. After 16.3 years of follow-up (from September 1986 through December 2002), there were 1375 cases of lymphatic malignancies, after exclusion of aforementioned cancer cases at baseline. Dietary data was complete and consistent for 1,277 cases, and 1,233 cases had complete data for covariables and were included in the analysis. Figure 1 shows the selection and exclusion steps that resulted in the numbers of cases and subcohort members that were available for analysis. Follow-up of the subcohort was nearly complete; only 1 male member of the subcohort was lost to follow-up.

### Acrylamide Intake Assessment

Data on dietary habits and potential confounding variables were collected at baseline by means of the NLCS food frequency questionnaire (FFQ). The FFQ contains questions on 150 foods, measuring frequency and portion size for most.

To estimate acrylamide intake, we used a database with acrylamide concentrations in foods on the Dutch market, provided by the Dutch Food and Consumer Product Safety Authority. In 2002, this authority analyzed the levels of acrylamide in various Dutch foods, such as bread, French fries, pastry, cake and Dutch spiced cake [26]. In 2005, the authority analyzed several foods to specifically accommodate the estimation of acrylamide intake of the NLCS cohort, such as various types of bread, specific types of cookies, cake and pastry, chocolate and chocolate milk, nuts and salty snacks, peanut butter and coffee. Acrylamide was measured in types of cookies which were known to be eaten most frequently by a population comparable with the NLCS, as was known from the development phase of the questionnaire. Bread was sampled and analyzed again in 2005, because the quantitation limit of the analytic method had decreased from 30 ppb in 2002 to 15 ppb in 2005. This change offered the opportunity to more accurately estimate the acrylamide intake via bread. To determine the acrylamide level for each food, the mean values of the acrylamide measurements per food were used, or, in case the concentrations were lower than the quantitation limit, a value one-half the quantitation limit. This database was recently validated in a study comparing estimated acrylamide content (using acrylamide data from the database) and measured acrylamide content of duplicate 24-hour diets [27]. This rendered a correlation coefficient of 0.82, which indicates that it is feasible to make a sound rank ordering of the acrylamide intake via a 24-hour meal using the mean acrylamide levels for individual foods in the database.

The acrylamide intake for each participant in the study was estimated by multiplying the acrylamide level of each food with the frequency of consumption and the portion size of that food, and summing up these values across all foods.

### Statistical Analysis

Acrylamide is included in the multivariable-adjusted models as a continuous variable per 10 µg per day intake of acrylamide as well as a categorical variable, to be able to investigate the dose-response relationship, where possible. For acrylamide to be modeled as a categorical variable, we required at least 100 cases for quintile categories in subgroup analyses or 60 cases for tertile categories. In case there were less than 60, but more than 20 cases in a subgroup, we analyzed acrylamide as a continuous variable only. Following these criteria, we thus analyzed multiple myeloma, diffuse large cell lymphoma, chronic lymphocytic leukemia, follicular lymphoma, Waldenstrom macroglobulinemia and immunocytoma in models with acrylamide as a continuous and categorical variable, and mantle cell lymphoma, and T-cell lymphoma in models with acrylamide as a continuous variable only. The numbers of other subtypes were too small for separate analyses.

Besides age and sex, the following variables were tested to assess potential confounding, based on the literature: body mass index (BMI), height, non-occupational physical activity, education level, vegetable and fruit intake, intake of several nutrients (such as fat and saturated fat, trans fatty acids, carbohydrates, fiber and niacin), alcohol consumption, smoking, reproductive factors (in women only; age at menarche, menopause and first childbirth, parity, and use of oral contraceptives and postmenopausal hormone treatment), and immune system-related diseases self-reported at baseline (such as hepatitis, tuberculosis and rheumatoid arthritis. Those variables that modified the hazard ratio of acrylamide (with a unit of 27 µg/day: the interval between the 10^th^ and 90^th^ percentile of acrylamide intake in the subcohort) by 10% or more for any endpoint were used in the final multivariable-adjusted model, which was subsequently applied to all endpoints. Some variables were tested for interaction on the basis of their ability to modify the activity of the enzyme CYP2E1. The variables concerned are age, BMI, diabetes, physical activity, and alcohol consumption [28], [29].

To test the proportional hazards assumption, models were run using scaled Schoenfeld residuals. Hazard ratios and corresponding 95% confidence intervals were obtained by performing Cox proportional hazards regression using STATA software (version 9; Stata Corp, College Station, TX) per subtype of lymphatic malignancies for men and women separately. Standard errors (SEs) were estimated by using the robust Huber-White sandwich estimator to account for additional variance introduced by sampling the subcohort from the cohort.

Smokers have on average three to four times higher levels of acrylamide-hemoglobin adducts (which is a marker of internal dose of acrylamide) than non-smokers. [30]. To preclude confounding through the exposure of cigarette smoke, subgroup analyses were performed for never-smokers.

Effect modification of the association between acrylamide intake and cancer by other variables was tested using Wald chi-square tests (by means of the testparm option in Stata). We thus tested whether there were any statistically significantly differences in the beta coefficients of acrylamide between the strata of the interaction variable.

To assess whether observed associations could be attributed to acrylamide intake or to the foods that contain the acrylamide, in separate analyses, acrylamide hazard ratios were adjusted for the five foods explaining most of the variance in acrylamide intake in our population, namely Dutch spiced cake, coffee, cookies, potato crisps and French fries.

All analyses were repeated without the first two years of follow-up to investigate protopathic bias.

## Results

The most outstanding differences between subcohort members and cases were observed for physical activity and family history between male subcohort members and male follicular lymphoma cases, and family history between female subcohort members and female mantle cell lymphoma cases (see table 2).

**Table 2:** Characteristics of cases and subcohort members according to sex in the Netherlands Cohort Study on diet and cancer, 1986–2002.

Variable^1^	Subcohort		CLL	DLCL	MM	FL		WMI	MCL	T-cell
**Men**										
N	2,191		139	165	172	44		54	41	36
*Dietary variables*										
Acrylamide intake (µg/d)	23 (12)		21 (11)	23 (13)	25 (13)	26 (16)		26 (17)	22 (10)	22 (11)
Acrylamide intake BW (µg/d per kg bw)	0.29 (0.16)		0.28 (0.15)	0.29 (0.17)	0.33 (0.18)	0.34 (0.21)		0.33 (0.21)	0.27 (0.13)	0.29 (0.15)
Coffee (g/d)	578 (290)		576 (304)	561 (265)	547 (233)	588 (295)		545 (209)	570 (296)	601 (283)
Dutch spiced cake (g/d)	4.1 (8.6)		3.2 (6.6)	4.2 (8.4)	5.5 (9.7)	8.9 (14.2)		6.4 (13.6)	3.8 (7.4)	3.1 (7.7)
Cookies (g/d)	14 (11)		14 (9)	13 (11)	16 (18)	12 (10)		15 (10)	16 (9)	17 (10)
Potato chips (g/d)	0.47 (1.72)		0.30 (1.01)	0.59 (3.16)	0.62 (2.54)	0.21 (0.57)		0.35 (0.71)	0.59 (1.84)	0.33 (1.02)
French fries (g/d)	7.2 (15.4)		6.3 (12.8)	7.7 (19.9)	9.4 (17.9)	4.8 (8.0)		9.1 (17.1)	4.6 (11.7)	5.4 (10.5)
Total energy intake (kcal/d)	2,162 (510)		2,208 (509)	2,133 (486)	2,194 (541)	2,164 (568)		2,239 (489)	2,171 (395)	2,180 (375)
Carbohydrate (g/d)	227 (66)		229 (61)	226 (61)	232 (65)	224 (56)		238 (66)	220 (44)	232 (48)
Saturated fat (g/d)	37 (12)		39 (13)	36 (12)	37 (12)	35 (12)		37 (11)	36 (10)	37 (10)
Trans unsaturated fatty acid (g/d)	3.3 (1.7)		3.6 (1.7)	3.5 (2.0)	3.6 (1.8)	2.9 (1.3)		3.4 (1.6)	3.1 (1.5)	3.2 (1.4)
Total fatty acids (g/d)	87 (27)		91 (27)	86 (25)	90 (29)	89 (34)		87 (25)	89 (23)	88 (22)
Mono unsaturated fat (g/d)	35 (12)		38 (12)	35 (11)	37 (13)	36 (16)		36 (10)	35 (10)	36 (10)
Poly unsaturated fat (g/d)	20 (10)		19 (8)	19 (9)	21 (10)	22 (14)		19 (9)	23 (11)	20 (8)
Fiber (g/d)	29 (9)		29 (9)	28 (9)	30 (9)	29 (9)		31 (8)	30 (9)	30 (8)
Alcohol (g/d)	15 (17)		14 (16)	14 (17)	12 (13)	12 (13)		19 (22)	17 (15)	12 (13)
Niacin (g/d)	15 (5)		16 (5)	15 (4)	15 (5)	16 (6)		16 (4)	15 (4)	16 (5)
*Non-dietary variables*										
Age (y)	61.3 (4.2)		62.1 (4.1)	62.0 (4.0)	61.8 (3.9)	61.1 (4.4)		61.8 (4.1)	62.0 (4.4)	61.1 (4.2)
BMI (kg/m^2^)	25.0 (2.6)		25.0 (2.4)	25.0 (2.4)	25.2 (2.7)	24.8 (2.7)		25.4 (2.3)	25.2 (2.0)	24.6 (2.6)
Height (m)	1.76 (0.07)		1.77 (0.06)	1.78 (0.07)	1.76 (0.07)	1.78 (0.08)		1.76 (0.07)	1.80 (0.06)	1.77 (0.08)
Non-occupational physical activity (min/d)	80 (68)		84 (84)	79 (68)	80 (62)	107 (96)		66 (52)	82 (85)	79 (72)
Family history of HM (%)	2.5		5.0	4.9	2.9	9.1		3.7	7.3	0.0
Cigarette smoking										
Never-smokers (%)	12.7		15.1	11.5	13.4	11.4		9.3	19.5	13.9
Former smokers (%)	51.6		55.4	47.9	59.3	38.6		59.3	58.5	41.7
Current smokers (%)	35.7		29.5	40.6	27.3	50.0		31.5	22.0	44.4
Smoking quantity (n cig./d)^2^	17 (11)		15 (11)	16 (10)	15 (10)	20 (12)		18 (15)	16 (11)	15 (8)
Smoking duration (y)^2^	34 (12)		34 (12)	34 (12)	32 (13)	35 (10)		32 (13)	33 (13)	36 (11)
Education										
Primary school (%)	25.0		24.6	25.8	22.2	27.9		24.1	17.5	8.3
Lower vocational school (%)	20.7		18.1	19.6	26.3	20.9		9.3	20.0	22.2
Intermediate vocational school (%)	35.6		40.6	31.9	33.3	32.6		31.5	40.0	50.0
Higher vocational school (%)	18.7		16.7	22.7	18.1	18.6		35.2	22.5	19.4
**Women**										
N	2247		68	104	161		49	35	18	20
*Dietary variables*										
Acrylamide intake (µg/d)	21 (12)		20 (11)	21 (11)	21 (13)		23 (18)	23 (15)	20 (14)	27 (19)
Acrylamide intake BW (µg/d per kg bw)	0.32 (0.19)		0.29 (0.17)	0.31 (0.17)	0.30 (0.19)		0.34 (0.26)	0.33 (0.20)	0.31 (0.25)	0.40 (0.30)
Coffee (g/d)	497 (245)		494 (241)	503 (202)	511 (234)		515 (258)	471 (285)	458 (271)	494 (172)
Dutch spiced cake (g/d)	5.7 (9.4)		4.9 (8.4)	5.4 (8.9)	5.2 (9.8)		7.9 (16.3)	8.2 (11.8)	6.1 (11.6)	10.0 (14.8)
Cookies (g/d)	14 (11)		14 (9)	14 (14)	15 (12)		13 (9)	15 (11)	14 (9)	15 (11)
Potato chips (g/d)	0.40 (1.93)		0.62 (3.15)	0.19 (0.76)	0.16 (0.46)		0.16 (0.52)	0.27 (0.94)	0.11 (0.28)	0.43 (1.08)
French fries (g/d)	4.0 (8.8)		1.7 (4.6)	3.1 (7.7)	4.0 (12.5)		2.8 (8.5)	3.4 (6.0)	1.4 (3.4)	5.7 (11.1)
Total energy intake (kcal)	1,683 (397)		1,762 (463)	1,634 (339)	1,711 (402)		1,747 (384)	1,708 (302)	1628 (370)	1,878 (468)
Carbohydrate (g/d)	179 (48)		190 (58)	174 (45)	184 (45)		183 (49)	184 (35)	165 (40)	205 (63)
Saturated fat (g/d)	30 (10)		31 (11)	30 (9)	30 (10)		32 (9)	29 (7)	29 (9)	35 (11)
Trans unsaturated fatty acid (g/d)	2.5 (1.2)		2.3 (0.9)	2.4 (0.9)	2.6 (1.3)		2.5 (0.9)	2.3 (0.9)	2.2 (0.9)	3.1 (1.4)
Total fatty acids (g/d)	69 (22)		71 (28)	68 (18)	70 (23)		70 (16)	69 (16)	70 (21)	77 (22)
Mono unsaturated fat (g/d)	28 (9)		29 (12)	27 (7)	28 (10)		28 (7)	28 (7)	29 (10)	31 (9)
Poly unsaturated fat (g/d)	15 (7)		15 (10)	15 (7)	15 (8)		14 (7)	17 (6)	16 (5)	16 (7)
Fiber (g/d)	25 (7)		26 (8)	26 (7)	27 (8)		26 (6)	27 (7)	26 (5)	26 (6)
Alcohol (g/d)	5.9 (9.5)		5.5 (9.6)	3.7 (7.2)	4.6 (7.7)		5.9 (10.7)	5.5 (8.9)	6.6 (8.5)	5.7 (8.6)
Niacin (g/d)	12 (3)		13 (5)	12 (3)	13 (4)		13 (3)	12 (3)	13 (3)	13 (3)
*Non-dietary variables*										
Age (y)	61.4 (4.3)		62.6 (4.0)	62.0 (4.6)	62.4 (4.2)		61.9 (4.3)	62.0 (4.2)	61.6 (4.4)	60.3 (4.3)
BMI (kg/m^2^)	25.1 (3.6)		25.1 (4.3)	24.6 (3.3)	25.6 (3.6)		15.2 (3.6)	25.0 (3.6)	23.9 (2.7)	24.9 (3.3)
Height (m)	1.65 (0.06)		1.67 (0.07)	1.66 (0.07)	1.66 (0.06)		1.66 (0.05)	1.67 (0.05)	1.66 (0.08)	1.65 (0.07)
Non-occupational physical activity (min/d)		64 (53)	67 (59)	62 (54)	70 (62)	62 (44)		76 (71)	69 (71)	52 (31)
Family history of HM (%)	3.2		2.9	4.8	5.6	6.1		0.0	11.1	5.0
Cigarette smoking										
Never-smokers (%)	58.4		66.2	65.4	67.7	57.1		57.1	50.0	65.0
Former smokers (%)	20.6		20.6	17.3	21.1	14.2		25.7	27.8	20.0
Current smokers (%)	21.0		13.2	17.3	11.2	28.6		17.1	22.2	15.0
Smoking quantity (n cig./d)^2^	11 (8)		15 (12)	12 (8)	11 (8)	11(7)		11 (5)	12 (7)	8 (11)
Smoking duration (y)^2^	28 (13)		27 (14)	29 (11)	26 (12)	30 (11)		30 (11)	23 (14)	24 (12)
Education										
Primary school (%)	33.5		29.4	35.0	34.4	27.1		34.3	22.2	35.0
Lower vocational school (%)	23.2		25.0	19.4	25.0	20.8		17.1	22.2	10.0
Intermediate vocational school (%)	34.5		38.2	39.8	34.4	43.8		40.0	44.4	45.0
Higher vocational school (%)	8.8		7.4	5.8	6.3	8.3		8.6	11.1	10.0

MM  =  multiple myeloma; DLCL  =  diffuse large cell lymphoma; CLL  =  chronic lymphocytic leukemia; FL  =  follicular lymphoma; WMI  =  Waldenström macroglobulinemia and immunocytoma; MCL  =  mantle cell lymphoma; T-cell  =  T-cell lymphomas; BW  =  bodyweight; HM  =  hematological malignancies.

1 Mean (standard deviation) or percentage.

2 Among former or current smokers.

As described elsewhere, coffee was overall the biggest source of acrylamide, but Dutch spiced cake was mainly responsible for the variation in acrylamide intake, and next most responsible were coffee, French fries, potato crisps, and cookies [31].

The hazard ratios for endpoints with more than 100 cases are presented in table 3. For multiple myeloma, there was an increased HR for the continuous acrylamide variable in all men (smokers and non-smokers combined) (HR per 10 µg acrylamide/day: 1.14; 95% CI: 1.01, 1.27), and a trend across the quintiles of acrylamide intake (*p* for trend  = 0.02). For never-smoking men, the HR for the continuous acrylamide intake was increased as well (HR per 10 µg acrylamide/day: 1.98; 95% CI: 1.38, 2.85). Unfortunately, the limited number of cases in this subgroup prohibited investigation of the dose-response relationship over categories of acrylamide intake. No association with multiple myeloma was observed in women, except for an increased HR in the 2^nd^ quintile in never-smoking women.

**Table 3:** Association between dietary acrylamide intake and the risk of multiple myeloma, diffuse large cell lymphoma, and chronic lymphocytic leukemia according to sex and smoking status; the Netherlands Cohort Study on diet and cancer, 1986–2002.^1^

	Acrylamide intake (per 10 µg/d)		Q1/T1	Q2	Q3/T2		Q4	Q5/T3	*P* for trend
Multiple myeloma									
All men									
Cases/py	170/28,981		32/5,656	20/5,661	35/5,899		34/5,833	49/5,933	
HR (CI 95%)^2^	1.15 (1.04–1.27)		1.00 (ref)	0.63 (0.36–1.13)	1.10 (0.67–1.82)		1.09 (0.66–1.82)	1.51 (0.94–2.41)	0.01
HR (CI 95%)^3^	1.14 (1.01–1.27)		1.00 (ref)	0.65 (0.36–1.16)	1.14 (0.67–1.94)		1.14 (0.67–1.94)	1.54 (0.92–2.58)	0.02
Never-smoking men									
Cases/py	23/3933								
HR (CI 95%)^2^	1.59 (1.24–2.03)		4	4	4		4	4	4
HR (CI 95%)^3^	1.98 (1.38–2.85)		4	4	4		4	4	4
All women									
Cases/py	153/32,296		25/6,305	41/6,586	34/6,286		23/6,630	30/6,489	
HR (CI 95%)^2^	0.99 (0.85–1.16)		1.00 (ref)	1.64 (0.98–2.74)	1.46 (0.86–2.49)		0.93 (0.52–1.67)	1.21 (0.70–2.09)	0.66
HR (CI 95%)^3^	0.92 (0.77–1.11)		1.00 (ref)	1.46 (0.85–2.49)	1.19 (0.67–2.12)		0.73 (0.39–1.37)	0.93 (0.50–1.73)	0.22
Never-smoking women									
Cases/py	102/19,005		13/4,058	32/3,866	19/3,414		16/3,903	22/3,765	
HR (CI 95%)^2^	1.08 (0.89–1.30)		1.00 (ref)	2.61 (1.34–5.08)	1.85 (0.90–3.83)		1.33 (0.63–2.81)	1.86 (0.92–3.76)	0.71
HR (CI 95%)^3^	1.01 (0.80–1.26)		1.00 (ref)	2.37 (1.19–4.73)	1.54 (0.72–3.29)		1.03 (0.46–2.31)	1.43 (0.68–3.02)	0.61
Diffuse large cell lymphoma									
All men									
Cases/py	159/28,981		32/5,656	28/5,661	35/5,899		34/5,833	30/5,933	
HR (CI 95%)^2^	1.02 (0.90–1.17)		1.00 (ref)	0.89 (0.53–1.50)	1.12 (0.68–1.84)		1.12 (0.67–1.85)	0.93 (0.56–1.56)	0.94
HR (CI 95%)^3^	1.04 (0.91–1.20)		1.00 (ref)	0.93 (0.54–1.59)	1.23 (0.74–2.04)		1.26 (0.74–2.17)	1.06 (0.61–1.86)	0.73
Never-smoking men									
Cases/py	19/3,933		4	4	4		4	4	4
All women									
Cases/py	100/32,296		17/6,305	17/6,586	24/6,286		24/6,630	18/6,489	
HR (CI 95%)^2^	0.98 (0.84–1.14)		1.00 (ref)	0.99 (0.50–1.98)	1.49 (0.78–2.84)		1.41 (0.73–2.69)	1.06 (0.54–2.09)	0.80
HR (CI 95%)^3^	1.02 (0.85–1.24)		1.00 (ref)	1.05 (0.51–2.15)	1.71 (0.87–3.36)		1.72 (0.84–3.50)	1.38 (0.63–3.02)	0.43
Never-smoking women									
cases/py	64/19,005		17/6,063		28/6,306			19/6,636	
HR (CI 95%)^2^	1.00 (0.81–1.22)		1.00 (ref)		1.67 (0.91–3.07)			1.05 (0.54–2.03)	0.73
HR (CI 95%)^3^	1.06 (0.83–1.36)		1.00 (ref)		1.79 (0.94–3.38)			1.27 (0.61–2.66)	0.94
Chronic lymphocytic leukemia									
All men^3^									
cases/py	134/28,981								
HR (CI 95%)^2^	0.94 (0.81–1.09)	5		5		5	5	5	5
HR (CI 95%)^3^	0.88 (0.74–1.04)	5		5		5	5	5	5
Never-smoking men									
cases/py	21/3,933								
HR (CI 95%)^2^	1.04 (0.76–1.40)	4		4		4	4	4	4
HR (CI 95%)^3^	1.12 (0.82–1.54)	4		4		4	4	4	4
All women^3^									
cases/py	66/32,296	24/9,743				19/11,168		23/11,385	
HR (CI 95%)^2^	0.99 (0.71–1.10)	1.00 (ref)				0.74 (0.40–1.37)		0.85 (0.47–1.53)	0.74
HR (CI 95%)^3^	0.83 (0.64–1.09)	1.00 (ref)				0.74 (0.38–1.43)		0.81 (0.42–1.57)	0.70
Never-smoking women									
cases/py	45/19,005								
HR (CI 95%)^2^	0.97 (0.75–1.25)	4		4		4	4	4	4
HR (CI 95%)^3^	0.95 (0.70–1.30)	4		4		4	4	4	4

1 HR  =  hazard ratio; CI  =  Confidence Interval; py  =  person years; Q  =  quintile; T  =  tertile. The number of cases and person-years are the numbers that resulted after listwise deletion of observations with missing values for the selected confounders. HRs were calculated by using Cox proportional hazards analysis.

2 Adjusted for age and sex.

3 Adjusted for age (years), sex, height (per 10 cm), education level, fiber (g/d), total fatty acids (g/d), trans unsaturated fatty acid (g/d), mono unsaturated fat (g/d), poly unsaturated fat (g/d), carbohydrates (g/d) and niacin (mg/d).

4 Insufficient number of cases for analyses with tertiles (N>60 required) or with acrylamide as a continuous variable (N>20 required).

5 Proportional hazards assumption not met; therefore results not presented.

Acrylamide was not associated with diffuse large cell lymphoma in any of the subgroups of men, women, or never-smoking men and women.

We observed decreased risks of chronic lymphocytic leukemia in both men and women for acrylamide as a continuous variable. In the subgroup of men, the proportional hazards assumption was violated in the quintile analysis. When we split the follow-up time at 2 years, at 8 years, or at 5 and 10 years, no clear associations between acrylamide intake and CLL risk in men were observed. For instance, in the first 8 years of follow-up, the hazard ratios for tertiles of acrylamide intake were 0.85 (95% CI: 045–1.63) and 0.80 (95% CI: 0.41–1.55) for the 2^nd^ and 3^rd^ tertile, respectively, with a p-trend of 0.50 (n  = 53). In the last 8.3 years the corresponding values were 1.10 (95% CI: 0.61–1.99) and 0.67 (0.34–1.31), with a p-trend of 0.19 (n  = 81). No association was seen in never-smokers.

Table 4 shows the hazard ratios for the continuous acrylamide variable for endpoints with less than 100 cases. The HR for acrylamide as a continuous variable for follicular lymphoma in all men was increased (HR per 10 µg acrylamide/day: 1.28; 95% CI: 1.03, 1.61), but not in all or never-smoking women.

**Table 4:** Association between continuously modeled dietary acrylamide intake (per 10 µg/d) and the risk of follicular lymphoma and Waldenstrom macroglobulinemia and immunocytoma (WMI); the Netherlands Cohort Study on diet and cancer, 1986–2002.^1^

	Follicular lymphoma	Waldenström macroglobulinemia and immunocytoma	Mantle cell lymphoma	T-cell lymphomas
All men				
Cases/py	42/28,981	54/28,981	38/28,981	35/28,981
HR per 10 µg/d (CI 95%)^1^	1.20 (0.98–1.47)	1.18 (0.96–1.44)	0.96 (0.76–1.21)	0.94 (0.70–1.25)
HR per 10 µg/d (CI 95%)^2^	1.28 (1.03–1.61)	1.18 (0.93–1.50)	1.06 (0.85–1.31)	0.94 (0.68–1.29)
Never-smoking men				
Cases/py	5/3,933	5/3,933	7/3,933	5/3,933
HR per 10 µg/d (CI 95%)^1^	3	3	3	3
HR per 10 µg/d (CI 95%)^2^	3	3	3	3
All women				
Cases/py	47/32,296	35/32,296	18/32,296	19/32,296
HR per 10 µg/d (CI 95%)^1^	1.12 (0.83–1.51)	1.13 (0.85–1.50)	3	3
HR per 10 µg/d (CI 95%)^2^	1.12 (0.80–1.57)	1.21 (0.88–1.66)	3	3
Never-smoking women				
Cases/py	27/19,005	20/19,005	9/19,005	12/19,005
HR per 10 µg/d (CI 95%)^1^	1.23 (0.91–1.67)	1.14 (0.78–1.68)	3	3
HR per 10 µg/d (CI 95%)^2^	1.18 (0.82–1.71)	1.27 (0.84–1.91)	3	3

HR  =  hazard ratio; CI  =  confidence interval; py  =  person years. The number of cases and person-years are the numbers that resulted after listwise deletion of observations with missing values for the selected confounders. HRs were calculated by using Cox proportional hazards analysis.

1 Adjusted for age and sex.

2 Adjusted for age (years), sex, height (per 10 cm), education level, fiber (g/d), total fatty acids (g/d), trans unsaturated fatty acid (g/d), mono unsaturated fat (g/d), poly unsaturated fat (g/d), carbohydrates (g/d) and niacin (mg/d).

3 Insufficient number of cases for analyses with acrylamide as a continuous variable (N>20 requiered).

We did not observe associations between acrylamide intake and the risk of Waldenström macroglobulinemia and immunocytoma in men or in women, or mantle cell lymphoma or T-cell-lymphoma in men. There were too few women in these latter two groups for meaningful analyses.

The results of the analyses of interactions between acrylamide and possible CYP2E1-influencing variables are presented in table 5 (men) and 6 (women) for multiple myeloma, diffuse large cell lymphoma and chronic lymphocytic leukemia. The numbers for other subtypes were too small for analysis of interaction.

**Table 5:** Acrylamide hazard ratios (and 95% CI) of multiple myeloma, diffuse large cell lymphoma and chronic lymphatic leukemia in **men** in strata of several covariables and *p* values for interaction: the Netherlands Cohort Study on diet and cancer, 1986–2002.

	MM			DLCL			CLL		
Interaction variable	N cases/person-years	HR per 10 µg AA/d	*P* for interaction	N cases/person-years	HR per 10 µg AA/d	*P* for interaction	N cases/person-years	HR per 10 µg AA/d	*P* for Interaction
Smoking status									
Never	23/3,933	1.92 (1.34–2.75)	0.02	19/3,933	1.03 (0.67–1.57)	0.05	21/3,933	1.06 (0.80–1.40)	0.45
Former	101/15,124	0.99 (0.82–1.19)		76/15,124	1.17 (0.97–1.42)		72/15,124	0.69 (0.53–0.90)	
Current	46/9,925	1.18 (0.96–1.44)		64/9,925	0.90 (0.74–1.09)		41/9,925	1.04 (0.79–1.36)	
Current smoking									
Smoking quantity (n cig./d)									
0	23/3,933	1.92 (1.34–2.75)	0.03	19/3,933	1.03 (0.67–1.57)	0.64	21/3,933	1.06 (0.80–1.40)	0.77
0 to <15	60/9,445	1.11 (0.92–1.34)		61/9,445	1.04 (0.85–1.28)		52/9,445	0.80 (0.62–1.03)	
≥15	82/13,935	1.04 (0.84–1.29)		73/13,935	1.13 (0.88–1.44)		52/13,935	0.86 (0.59–1.24)	
Smoking duration (y)									
0	23/3,933	1.92 (1.34–2.75)	0.02	19/3,933	1.03 (0.67–1.57)	0.61	21/3,933	1.06 (0.80–1.40)	0.30
0 to <30	56/8,513	1.00 (0.81–1.23)		48/8,513	1.04 (0.83–1.31)		39/8,513	0.60 (0.40–0.89)	
>30	89/1,6117	1.12 (0.95–1.31)		91/16,117	1.06 (0.88–1.28)		73/16,117	0.97 (0.76–1.23)	
Age									
Ever and never-smokers									
55–59	58/11,922	1.03 (0.83–1.27)	0.48	50/11,922	0.93 (0.65–1.31)	0.07	43/11,922	0.96 (0.76–1.22)	0.37
60–64	64/10,059	1.23 (0.97–1.55)		62/10,059	1.24 (0.99–1.55)		50/10,059	0.81 (0.57–1.15)	
65–69	48/7,000	1.19 (0.99–1.43)		47/7,000	0.84 (0.60–1.20)		41/7,000	0.83 (0.56–1.24)	
Never-smokers									
55–59	7/1,602	2	2	3			6/1,602	2	2
60–64	9/1,285	2					5/1,285	2	
65–69	7/1,045	2					10/1,045	0.92 (0.42–2.01)	
BMI (kg/m^2^)									
Ever and never-smokers									
<20	2/520	2	2	2/520	2	2	4/520	2	2
≥20–25	82/14,885	1.26 (1.10–1.44)		76/14,885	1.08 (0.90–1.29)		59/14,885	0.85 (0.66–1.10)	
>25	85/13,380	0.88 (0.70–1.09)		79/13,380	1.03 (0.82–1.31)		70/13,380	0.91 (0.73–1.14)	
Never-smokers									
<20	0/64	2	2	3			1/64	2	2
≥20–25	15/2,130	2.09 (1.43–3.05)					13/2,130	1.00 (0.59–1.70)	
>25	7/1,706	2					7/1,706	2	
Diabetes									
Ever and never-smokers									
No	162/28,192	1.14 (1.02–1.29)	2	156/28,192	1.05 (0.91–1.21)	2	128/28,192	0.88 (0.74–1.04)	2
Yes	8/790	2		3/790	2		6/790	2	
Never-smokers									
No	22/3,840	1.94 (1.34–2.82)	2	3			20/3,840	1.14 (0.82–1.59)	2
Yes	1/93	2					1/93	2	
Physical activity (min/d)									
Ever and never-smokers									
<30	26/4,503	1.11 (0.85–1.45)	0.85	38/4,503	0.84 (0.61–1.16)	0.40	29/4,503	0.72 (0.50–1.04)	0.57
30–60	52/9,341	1.21 (0.94–1.56)		44/9,341	1.07 (0.84–1.36)		38/9,341	0.90 (0.65–1.24)	
61–90	38/5,467	0.99 (0.75–1.31)		275,467	1.09 (0.67–1.76)		25/5,467	0.84 (0.50–1.41)	
>90	53/9,203	1.14 (0.94–1.39)		48/9,203	1.13 (0.90–1.41)		40/9,203	0.98 (0.74–1.31)	
Never-smokers									
<30	3/656	2	2	3			4/656	2	2
30–60	9/1,116	2					6/1,116	2	
61–90	5/873	2					3/873	2	
>90	6/1,181	2					8/1,181	2	
Alcohol intake (g/d)									
Ever and never-smokers									
0	14/4,004	0.78 (0.34–1.77)	0.53	25/4,004	1.12 (0.93–1.34)	0.72	12/4,004	1.08 (0.69–1.71)	0.56
>0–5	46/6,084	1.29 (1.02–1.64)		34/6,084	1.01 (0.62–1.66)		35/6,084	0.92 (0.61–1.41)	
>5	109/18,553	1.14 (0.98–1.33)		99/18,553	0.99 (0.82–1.20)		85/18,553	0.81 (0.65–1.01)	
Never-smokers									
0	2/874	2	2	3			2/874	2	2
>0–5	11/1,122	1.81 (1.17–2.80)					6/1,122	2	
>5	10/1,921	2.28 (1.28–4.06)					13/1,921	0.96 (0.56–1.64)	

Abbreviations: HR  =  hazard ratio; CI  =  confidence interval; AA/d  =  acrylamide per day, MM  =  multiple myeloma; CLL  =  chronic lymphatic leukemia; DLCL  =  diffuse large cell lymphoma.

1 Adjusted for age, sex, height (per 10 cm), education level, fiber (g/d), total fatty acids (g/d), trans unsaturated fatty acid (g/d), mono unsaturated fat (g/d), poly unsaturated fat (g/d), carbohydrates (g/d) and niacin (mg/d).

2 Insufficient number of cases for analyzing interaction.

3 This subgroup was not analyzed at all, due to insufficient number of cases (n<20).

**Table 6:** Acrylamide hazard ratios (and 95% CI) of multiple myeloma, diffuse large cell lymphoma and chronic lymphatic leukemia in **women** in strata of several covariables and *p* values for interaction: the Netherlands Cohort Study on diet and cancer, 1986–2002.

	MM			DLCL			CLL		
Interaction variable	N cases/person-years	HR per 10 µg AA/d	*P* for interaction	N cases/person-years	HR per 10 µg AA/d	*P* for interaction	N cases/person-years	HR per 10 µg AA/d	*P* for interaction
Smoking status									
Never	102/19,005	1.02 (0.80–1.26)	0.24	64/19,005	1.06 (0.83–1.36)	0.36	45/19,005	0.93 (0.68–1.27)	2
Former	33/6,716	0.74 (0.49–1.12)		18/6,716	1.29 (0.86–1.91)		13/6,716	0.72 (0.41–1.28)	
Current	18/6,574	0.67 (0.31–1.44)		18/6,574	0.49 (0.19–1.24)		8/6,574	2	
Current smoking									
Smoking quantity (n cig./d)									
0	102/19,005	1.01 (0.80–1.26)	0.23	64/19,005	1.06 (0.83–1.36)	0.83	45/19,005	0.93 (0.68–1.27)	2
0 to <15	37/8,238	0.63 (0.39–1.02)		24/8,238	1.06 (0.72–1.55)		11/8,238	0.79 (0.44–1.41)	
≥15	14/4,501	0.94 (0.49–1.83)		12/4,501	0.83 (0.50–1.36)		9/4,501	2	
Smoking duration (y)									
0	102/19,005	1.01 (0.80–1.26)	0.18	64/19,005	1.06 (0.83–1.36)	0.83	45/19,005	0.93 (0.68–1.27)	2
0 to <30	28/6,405	0.66 (0.39–1.12)		15/6,405	1.05 (0.60–1.87)		9/6,405	2	
≥30	22/6,530	0.79 (0.42–1.49)		21/6,530	0.98 (0.64–1.50)		11/6,530	0.66 (0.31–1.43)	
Age									
Ever and never-smokers									
55–59	43/13,090	0.89 (0.68–1.16)	0.66	36/13,090	1.03 (0.76–1.38)	0.14	17/13,090	0.44 (0.16–1.23)	0.27
60–64	57/10,768	1.00 (0.74–1.36)		29/10,768	1.41 (0.96–2.07)		23/10,768	0.96 (0.62–1.49)	
65–69	53/8,437	0.88 (0.60–1.30)		35/8,437	0.77 (0.56–1.06)		26/8,437	0.96 (0.70–1.32)	
Never-smokers									
55–59	27/6,852	0.90 (0.67–1.22)	0.87	20/6,852	1.05 (0.74–1.51)	0.22	6/6,852	2	2
60–64	38/6,424	0.99 (0.65–1.52)		18/6,424	1.35 (0.82–2.22)		17/6,424	0.80 (0.45–1.42)	
65–69	37/5,729	1.13 (0.73–1.74)		26/5,729	0.82 (0.56–1.21)		22/5,729	1.01 (0.69–1.49)	
BMI (kg/m^2^)									
Ever and never-smokers									
<20	4/1,520	2	2	4/1,520	2	2	9/1,520	2	2
≥20–25	66/16,362	0.83 (0.61–1.14)		51/16,362	1.03 (0.79–1.35)		30/16,362	0.67 (0.41–1.09)	
>25	80/14,279	1.04 (0.81–1.32)		45/14,279	1.04 (0.78–1.38)		27/14,279	0.99 (0.68–1.45)	
Never-smokers									
<20	1/658	2	2	1/658	2	2	5/658	2	2
≥20–25	42/9,189	0.91 (0.60–1.37)		33/9,189	1.03 (0.71–1.51)		19/9,189	0.81 (0.43–1.52)	
>25	58/9,119	1.11 (0.85–1.45)		30/9,119	1.04 (0.76–1.43)		21/9,119	1.12 (0.75–1.69)	
Diabetes									
Ever and never-smokers									
No	148/31,252	0.90 (0.75–1.07)	2	97/31,252	1.03 (0.85–1.25)	2	63/31,252	0.84 (0.64–1.11)	2
Yes	5/1,044	2		3//1,044	2		3/1,044	2	
Never-smokers									
No	98/18,264	0.96 (0.77–1.19)	2	63/18,264	1.06 (0.82–1.37)	2	42/18,264	0.97 (0.71–1.34)	2
Yes	4/742	2		1/742	2		3/742	2	
Physical activity (min/d)									
Ever and never-smokers									
<30	34/7,170	0.54 (0.35–0.83)	0.42	26/7,170	1.16 (0.86–1.58)	0.47	15/7,170	0.74 (0.45–1.22)	0.16
30–60	52/9,977	1.07 (0.79–1.45)		30/9,977	1.12 (0.82–1.53)		22/9,977	0.90 (0.57–1.43)	
61–90	32/7,236	1.01 (0.71–1.43)		21/7,236	0.95 (0.59–1.53)		16/7,236	0.75 (0.39–1.44)	
>90	31/7,089	1.10 (0.69–1.77)		21/7,089	0.78 (0.52–1.16)		13/7,089	1.05 (0.58–1.89)	
Never-smokers									
<30	23/4,493	0.49 (0.25–0.95)	0.26	15/4,493	1.24 (0.82–1.86)	0.83	12/4,493	0.79 (0.43–1.45)	2
30–60	36/6,215	1.36 (0.97–1.90)		21/6,215	1.17 (0.81–1.71)		15/6,215	1.10 (0.68–1.77)	
61–90	21/3,784	1.06 (0.71–1.57)		15/3,784	0.70 (0.31–1.57)		11/3,784	1.10 (0.58–2.09)	
>90	20/3,948	1.03 (0.51–2.09)		12/3,948	0.92 (0.54–1.55)		7/3,948	2	
Alcohol intake (g/d)									
Ever and never-smokers									
0	40/9,793	0.92 (0.67–1.28)	0.81	35/9,793	1.01 (0.72–1.41)	0.62	21/9,793	0.89 (0.56–1.39)	0.69
>0–5	65/11,351	0.95 (0.69–1.32)		45/11,351	0.98 (0.72–1.35)		21/11,351	0.60 (0.37–0.97)	
>5	39/9,812	0.94 (0.66–1.34)		18/9,812	1.15 (0.84–1.59)		21/9,812	1.00 (0.62–1.63)	
Never-smokers									
0	28/6,975	0.88 (0.59–1.31)	0.78	21/6,975	0.76 (0.46–1.26)	2	18/6,975	0.91 (0.52–1.57)	2
>0–5	51/7,410	1.13 (0.79–1.61)		33/7,410	1.21 (0.84–1.74)		16/7,410	0.62 (0.36–1.06)	
>5	17/3,692	1.05 (0.62–1.77)		8/3,692	2		8/3,692	2	

Abbreviations: HR  =  hazard ratio; CI  =  confidence interval; AA/d  =  acrylamide per day, MM  =  multiple myeloma; CLL  =  chronic lymphatic leukemia; DLCL  =  diffuse large cell lymphoma.

1 Adjusted for age, sex, height (per 10 cm), education level, fiber (g/d), total fatty acids (g/d), trans unsaturated fatty acid (g/d), mono unsaturated fat (g/d), poly unsaturated fat (g/d), carbohydrates (g/d) and niacin (mg/d).

2 Insufficient number of cases.

In the analyses of multiple myeloma, smoking status modified the acrylamide-associated risk in men. Never-smokers had a higher acrylamide-associated risk of multiple myeloma (HR per 10 µg acrylamide/day: 1.92 (95% CI: 1.34, 2.75; *p* for interaction  = 0.02)) than former or current smokers, which was also reflected by the interaction with smoking quantity and duration. Although there was no statistically significant interaction with alcohol, we observed an increased acrylamide-associated risk of multiple myeloma in never-smoking men with the highest (>5 g/day) intake of alcohol (HR 2.28 (95% CI: 1.28, 4.06) For diffuse large cell lymphoma, smoking status modified the acrylamide-associated risk in men, with former smokers having the highest acrylamide-associated risk (HR per 10 µg acrylamide/day: 1.17 (95% CI: 0.97, 1.42); *p* for interaction  = 0.05), but not in women (*p* for interaction  = 0.36).

For chronic lymphocytic leukemia, there was no interaction between acrylamide intake and any of the CYP2E1-influencing variables.

In sensitivity analyses, the associations between acrylamide and the endpoints of lymphatic malignancies did not change after exclusion of cases diagnosed during the first two years of follow-up. Although the HRs of acrylamide intake were slightly attenuated after additional adjustment for coffee, and increased after adjustment for Dutch spiced cake, the results did not change importantly. The results also did not change when adjusted for the other foods that contribute most to the variance in acrylamide intake, which were cookies, French fries and potato crisps. All of these foods were themselves not associated with the risk of lymphatic malignancies (results not shown).

At the time of the analyses described in this paper, our data on lymphatic malignancies were not classified according to the InterLymph classification [32]. We have checked how our classification of the cases corresponds to the InterLymph classification for the types of lymphatic malignancies that we observed associations with acrylamide for (multiple myeloma and follicular lymphoma in men). There were no (multiple myeloma) or minor differences (n  = 1 for follicular lymphoma) between the two classifications. When we left out the male case that was a follicular lymphoma case in our dataset, but that would have been a chronic/small lymphocytic leukemia/lymphoma case in the InterLymph classification, the hazard ratios for acrylamide were virtually unchanged. A major difference between the WHO classification and the InterLymph classification lies in the way lymphomas with morphology code M9675 are classified. In the WHO classification, they are grouped under diffuse large cell lymphoma, but not all M9675 lymphomas are large cell lymphomas and some are T-cell lymphomas. We have repeated the analysis of the diffuse large cell lymphoma group excluding the M9675 codes (n  = 27 men, 12 women) (which then renders the group of diffuse large B-cell lymphomas according to the InterLymph recommendations) and the results were essentially unchanged.

## Discussion

This prospective cohort study is, to our knowledge, the first epidemiological study to investigate the association between dietary acrylamide intake and the risk of specific histological subtypes of lymphatic malignancies. Because of this, the results of this study are challenging, but should be interpreted cautiously. We observed a positive association for multiple myeloma in all men and never-smoking men, and for follicular lymphoma in all men.

In the Finnish ATBC Study, no association was observed between dietary acrylamide intake and the risk of lymphomas in male smokers [17]. In that study, no analyses were done for histological subtypes of lymphatic malignancies, and therefore an association with a specific type of lymphatic malignancy might have been obscured. In addition, when studying the link between dietary acrylamide intake and cancer risk, it is better to study non-smokers as a subgroup, because cigarette smoke is a much more important source of acrylamide than diet is and it might therefore blur the association between acrylamide through diet and cancer risk.

Possible risk factors for lymphatic malignancies, such as height, overweight, hormones and nutrients, have shown contradictory results in epidemiological studies [20], [25]. Although there is thus no strong epidemiological evidence for risk factors for lymphatic malignancies, in the present study we checked the confounding potential of a broad range of possible risk factors for lymphatic malignancies and cancer in general. Human immunodeficiency virus (HIV) infection has been associated with an increased risk of lymphatic malignancies [20]. Data on the prevalence of HIV in our study population was not available, but the prevalence was likely low, considering the age segment of our population. We were able to check for other immune system-related diseases, such as asthma and tuberculosis, but these diseases were not found to be confounders for the association between acrylamide intake and the risk of lymphatic malignancies.

The present study has some limitations that should be discussed. The associations between dietary acrylamide intake and multiple myeloma in never-smoking men, and the association for follicular lymphoma in all men were based on analyses with a small number of cases. This makes it likely that some of the observed associations are spurious. Therefore, these results should be interpreted cautiously. Moreover, we analyzed associations in many subtypes of lymphatic malignancies and for several subgroups within each subtype, which makes it likely that chance findings have occurred. The same applies to the subgroup analyses that were done to investigate interaction with CYP2E1-influencing variables. However, the indications for possible interaction with smoking and alcohol are intriguing, although based on analyses with a small number of cases, as both smoking and alcohol intake were inversely associated with the glycidamide to acrylamide hemoglobin adduct ratio in a cross-sectional population study [33].

In addition, this study has some limitations regarding acrylamide intake assessment. Firstly, within foods, acrylamide levels vary greatly, which leads to non-differential misclassification of acrylamide intake when assigning a single mean acrylamide value to a food, which most likely biases risk estimates towards null. This means that true risks, if any, are probably greater than the risks presented here. Moreover, a recent study has shown that it is feasible to make a sound rank ordering of the acrylamide intake via a 24-hour meal using the mean acrylamide levels used in the NLCS study for individual foods. [27]. Secondly, the acrylamide values in our food database were derived from foods that were sampled in 2002 and 2005. They may not be completely representative of the foods that were on the market in 1986. Thirdly, the FFQ did not provide information on which foods were prepared at home and how this was done. Both aspects too will have resulted in some non-differential misclassification of the intake, which will then most likely have led to underestimation of the true risks. Despite the fact that the use of an FFQ has limitations for the assessment of dietary acrylamide exposure, as is extensively discussed elsewhere [34], it is the only feasible way to assess dietary acrylamide intake over a long period of time in a large study population.

Although we have no direct data for acrylamide itself, the reproducibility and validity of the NLCS FFQ for acrylamide can to some extent be derived from nutrients that are correlated to acrylamide, namely carbohydrates and dietary fiber. The decline of the correlation between the baseline questionnaire and the questionnaire administered after 5 years of follow-up was 0.07 on average among the nutrients that were tested. This indicates that, although the questionnaire was administered only once, it characterizes long-term food intake for over a period of at least five years [35]. As for validity, the correlation coefficients between the FFQ and a dietary record method were 0.77 for carbohydrates and 0.74 for fiber. For the food groups potatoes, bread, and cakes and cookies, Spearman correlation coefficients were 0.74, 0.80 and 0.65, respectively [36].

The large study size and the prospective nature of this NLCS are important strengths of this study. Selection bias is unlikely to occur, as the follow-up of the participants was complete. Due to the prospective design of the study, recall bias is absent. In addition, we were able to obtain a dietary acrylamide intake estimate representative for the Dutch study population, by estimating acrylamide levels in several batches of various Dutch food products that were specific for the population under study. The large study size enabled us to study specific histological subtypes of lymphatic malignancies that differ in their etiology and, as indicated by this study, may differ regarding their association with dietary acrylamide intake.

Recent analyses within the NLCS, the Nurses’ Health Study, and a Danish cohort study [11], [12], [13], [14] showed a positive association for endometrial, ovarian, and estrogen receptor-positive breast cancer, suggesting that disturbance of sex hormonal balances may be a mechanism of acrylamide carcinogenesis, which can also be suggested based on the rat carcinogenicity assays [7], [8]. Although it cannot be concluded from the present study, hormonal imbalances might be a mechanism of acrylamide carcinogenesis for lymphatic malignancies as well. Men have a higher incidence of lymphatic malignancies than women [20], but the reasons for this higher incidence are not known. Sex hormones have been shown to influence the immune system [37] and may thus be at the basis of this observed difference. Estrogen receptor expression in lymphocytes suggests that estrogen bioavailability may be relevant to the pathogenesis of lymphomas [38]. For multiple myeloma, studies investigated the mechanism of anti-estrogens (AEs), and showed that AEs inhibit cell cycle progression of malignant multiple myeloma cells and/or induce apoptosis in these cells [39]. Other studies suggest that hormone-related and reproductive factors are involved in the etiology of lymphatic malignancies [40], [41], [42], and in a different way for men and women [43], but the results are inconsistent.

This is the first epidemiological study to investigate the association between dietary acrylamide intake and the risk of lymphatic malignancies. It provides indications that acrylamide may increase the risk of multiple myeloma and follicular lymphoma, but on the basis of the present study alone, we cannot conclude whether these results reflect true biological effects or are chance findings. We recommend that this possible modifiable risk factor for lymphatic malignancies is investigated in other prospective studies.
